# Comparative Evaluation of Marginal Adaptation and Fracture Strength of Different Ceramic Inlays Produced by CEREC Omnicam and Heat-Pressed Technique

**DOI:** 10.1155/2018/5152703

**Published:** 2018-04-26

**Authors:** F. D. Oz, S. Bolay

**Affiliations:** Department of Restorative Dentistry, Faculty of Dentistry, Hacettepe University, Altindag, Ankara, Turkey

## Abstract

**Objective:**

The aim of this in vitro study was to evaluate marginal adaptation and fracture strength of inlays produced by CEREC Omnicam using different types of blocs and heat-pressed technique. Methods: Seventy-five extracted human mandibular molars were divided randomly into 5 groups (*n*=15). 60 molars in four groups received MOD inlay preparations. Experimental groups were CO: Intact teeth, EC: IPS e.max CAD and CEREC, LU: Lava Ultimate and CEREC, EL: IPS Empress CAD and CEREC, EP: IPS Empress Esthetic ingots and heat-pressed technique. Marginal gap measurements were taken with a stereomicroscope. Restorations were cemented with Variolink N and stored in distilled water at 37°C for 24 hours. All samples were subjected to thermocycling. The fracture strength of specimens was determined at a 0.5 mm/min crosshead speed until fracture. Fracture modes were determined. Statistical analyses were performed using one-way analysis of variance for fracture strength data and Kruskal–Wallis for marginal gap data (*p*=0.05).

**Results:**

The mean marginal gap size of EC, LU, EL, and EP were 33.54 *µ*m, 33.77 *µ*m, 34.23 *µ*m, and 85.34 *µ*m, respectively. EP had statistically higher values than other groups. The fracture strength values were significantly higher in the intact teeth group (3959,00 ± 1279,79 N) than those of restored groups EC (2408,00 ± 607,97 N), LU (2206,73 ± 675,16), EL (2573.27 ± 644,73) ve EP (2879,53 ± 897,30).

**Conclusion:**

Inlays fabricated using CEREC Omnicam demonstrated better marginal adaptation than inlays produced with heat-pressed technique, whereas fracture strength values of inlays fabricated with different type of blocks using CEREC Omnicam exhibited similarity to those fabricated with heat-pressed technique.

## 1. Introduction

Multiple parameters influence the longevity of dental restorations such as material properties, patient's health status, dentist's experience, and fabrication methods [1]. Restorations generated with indirect methods have desirable mechanical properties, and they are produced under slightly ideal in vitro conditions [2]. Additionally, patients' increasing demand for tooth-colored posterior restorations has stimulated the improvement of indirect methods and materials for posterior esthetic restorations [1]. Heat-pressed technique is one of the frequently used methods for making ceramic restorations. Leucite-reinforced glass ceramic IPS Empress System was manufactured with heat-pressed technique for the intention of restoring single units, including esthetic inlays [3]. Industrially fabricated machinable ceramics are produced for technician use in laboratory and clinician use for chair-side applications. Digital systems such as computer aided design and computer aided manufacturing (CAD/CAM) have evolved as an alternative for high-temperature injection molding using the lost-wax technique [4]. Moreover, with advancements in material sciences, ceramic restorations have shown better results to fulfil mechanical and esthetic necessities for patients [5]. Restorations fabricated using industrially made CAD/CAM ceramic blocks have remarkably improved mechanical properties such as reduction in cracks and voids in comparison with restorations produced in laboratory [6]. A wide collection of ceramic materials has been available for both CAD/CAM technology and heat-pressed technique, ranging from relatively weak leucite glass ceramic to high-strength lithium disilicate glass ceramic [7]. Different ceramic blocs are used for CAD/CAM technology such as IPS e.max CAD (Ivoclar Vivadent AG, Liechtenstein), a lithium disilicate improved glass-ceramic material with a relatively high fracture strength [8] and Lava Ultimate (3M-ESPE, St Paul, USA), a resin nanoceramic with an elastic modulus value similar to dentin [9]. Material properties can be an important factor for fracture strength [10]. Furthermore, the fracture strength of ceramic restorations is also influenced by elastic modulus [11].

Marginal adaptation is also crucial for ceramic inlay restorations to avoid resin cement wear and plaque accumulation. Marginal gap formations at restorations exposes resin cement to the oral environment leading to resin cement wear. Marginal discrepancies cause debris and food to act as potential irritants which might induce secondary caries and devitalization of the pulp [12]. Moreover, an unsuitable fit of the restoration cannot be well supported by the remaining tooth substance and influences the longevity of the restoration [13]. One of the most significant advances in dentistry has been the introduction of CAD/CAM systems. Popularity of these systems has increased significantly in the last decade due to the simplicity of their application [14]. Some studies which examined the marginal adaptation of different ceramic inlay systems have shown acceptable results [15, 16].

In the dental field, to improve impression techniques, lately a powder-free 3D oral scanning camera (CEREC Omnicam) was introduced to produce more precise teeth scans. However, the marginal adaptation of ceramic mesio-occlusal-distal (MOD) inlays produced by the heat-pressed technique has not been compared to those produced by the CEREC Omnicam CAD/CAM systems. Therefore, the aim of this study was to evaluate and compare the marginal adaptation and the fracture strength of MOD ceramic inlay restorations fabricated by CEREC Omnicam CAD/CAM system and heat-pressed technique. Also different types of ceramic blocs were compared in the means of fracture strength for ceramic inlays fabricated by CEREC Omnicam system.

## 2. Materials and Methods

In this in vitro study, 75 extracted, caries-free human mandibular third molars with similar buccolingual and mesiodistal dimensions were selected. Ethics Committee approval (GO 14/138-16) was obtained for the extracted human teeth. An electronic digital caliper (Absolute Digimatic Mitutoyo, Tokyo, Japan) were used for measurement. Teeth were stored in distilled water after calculus and soft-tissues were removed with a hand scaler and cleaned using a rubber cup. Teeth were examined under magnifying glasses (Hires 2.5, Orascoptic, CA, USA) to detect any preexisting defects. Only intact, noncarious, unrestored teeth were included in the study. These teeth were randomly divided into four groups (*n*=15). The root of each tooth was embedded in an autopolymerizing acrylic resin (Meliodent, Heraeus Kulzer GmbH, Hanau, Germany) up to 2 mm below the cementoenamel junction. Experimental groups were as follows:

  CO: intact teeth, no treatment (control group)
  EC: teeth restored with lithium disilicate glass-ceramic (IPS e.max CAD, Ivoclar Vivadent AG) using CEREC Omnicam
  LU: teeth restored with resin nanoceramic (Lava Ultimate, (3M ESPE, Seefeld, Germany) using CEREC Omnicam
  EL: teeth restored with leucite-reinforced glass ceramic (IPS Empress CAD, Ivoclar Vivadent AG) using CEREC Omnicam
  EP: teeth restored with leucite-reinforced glass ceramic (IPS Empress Esthetic, Ivoclar Vivadent AG) and hot pressed technique

Teeth in EC, LU, EL, and EP groups first received a standardized mesio-occlusal-distal preparation with the geometry of an inlay cavity using a straight fissure flat-ended diamond bur (Diatech Dental, Coltene-Whaledent, Altstatten, Switzerland) on a high-speed handpiece with water spray cooling. Diamond burs were used for five preparations, and a new bur was used after 5 specimen. Mesial and distal finishing lines of the proximal boxes were 1 mm above the cementoenamel junction and the proximal boxes had 2 mm width mesiodistally. Pulpal floors were prepared flat, and angles were rounded. The width of the occlusal cavity was designed to prepare 1/3 of that of the tooth, and the occlusal depth was prepared to 3 mm from the occlusal margin. The occlusogingival dimension of the proximal box was approximately 4.0 to 4.5 mm, depending on the length of the crown. A 6° divergence of the walls of the occlusal and proximal boxes was prepared using a tapered diamond with a convergence angle of 6°.

### 2.1. Fabrication of Inlay Restorations with CEREC Omnicam CAD/CAM System

**Figure 1:**
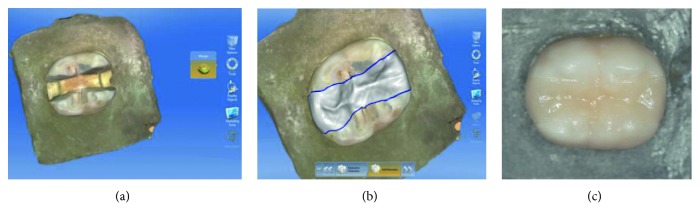
(a) Digital impression of a preparation in CEREC Omnicam. (b) Restoration design of Lava Ultimate restoration. (c) Lava Ultimate inlay restoration after cementation.

In the EC, LV, and EL groups, inlays were fabricated by CEREC Omnicam (Sirona Dental System, GmbH, Bensheim, Germany). Digital impressions were taken without powder application using CEREC SW 4.2.3 software. Following impressions and design (Figures 1(a) and 1(b)), occlusal thicknesses of restorations were checked using “cursor details” tools of the programme. It was ensured that all occlusal thicknesses were between 2.6 and 2.8 mm before milling procedure. Restorations from group EC were designed and milled with from presintered lithium disilicate glass-ceramic blocks (IPS e.max CAD, Ivoclar Vivadent, Schaan, Liechtenstein). Crystallization of IPS e.max CAD restorations was performed in Progmat P310 (Ivoclar Vivadent, Schaan, Liechtenstein) furnace after the milling procedure following the manufacturer's instruction. The temperature was 840°C, and the dwell time was 7 min. The restorations were then glazed with IPS e.max Ceram Glaze Liquid and Paste (Ivoclar Vivadent, Schaan, Liechtenstein). A single-glaze firing was performed in Progmat P310 (Ivoclar Vivadent, Schaan, Liechtenstein) furnace at 840°C with a dwell time of 3 min. Restorations from group EL were designed and fabricated from leucite-reinforced glass ceramic (IPS Empress CAD, Ivoclar Vivadent AG). The restorations were then glazed with IPS Empress CAD Universal Glaze Stain Liquid and Paste (Ivoclar Vivadent, Schaan, Liechtenstein). A single-glaze firing was performed in Progmat P310 (Ivoclar Vivadent, Schaan, Liechtenstein) furnace at 840°C with a dwell time of 3 min. On the other hand, LU restorations received a sandblasting treatment to the internal surfaces with 50 *μ*m alumina particles at an air pressure of 30 PSI. Finishing and polishing procedure of LU group was conducted with Sof-Lex discs (3M ESPE, St. Paul, USA, Batch number 5082S).

### 2.2. Fabrication of Inlay Restorations with Heat-Pressed Technique

A pressable leucite-reinforced glass-ceramic material was used in group EP (IPS Empress Esthetic, Ivoclar Vivadent AG). Impressions were taken using an elastometic material (Virtual Putty and Virtual Light Body, Ivoclar Vivadent, Schaan, Liechtenstein). After preparing stone dies, die spacer was applied, and wax models were fabricated according to appropriate anatomic functional form of each tooth. The wax models were invested in [SheraFina 2000] (SHERA Werkstoff-Technologie GmbH & Co., Lemförde, Germany, Batch number: 30894) investment material. The investment ring was heated at 1060°C for 60 min for the burn-out of the wax analog, and the ingots were pressed into the investment mold using a Programat EP 5000 (Ivoclar Vivadent, Schaan, Liechtenstein) furnace following the manufacturer's instructions. The press temperature was 1075°C, and the dwell time was 23 min. All restorations' occlusal thicknesses were checked using a digital caliper. If a restorations' thickness was not between 2.6 and 2.8, the procedure was repeated for standardization. Finally, a single-glaze firing was performed with Programat EP 5000 (Ivoclar Vivadent, Schaan, Liechtenstein) furnace using IPS Empress Universal Glaze and Stain Liquid and Paste (Ivoclar Vivadent, Schaan, Liechtenstein).

### 2.3. Marginal Gap Evaluation

**Figure 2:**
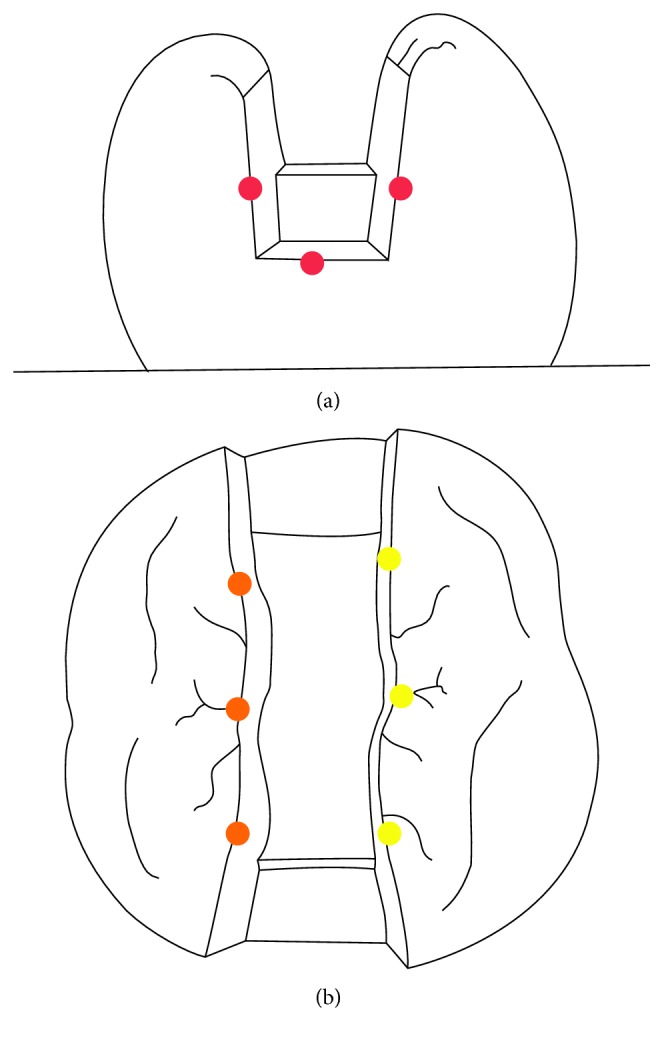
Marginal gap measurement points. (a) Location of measuring points mesially and distally. (b) Location of measuring points on the occlusal aspect.

**Figure 3:**
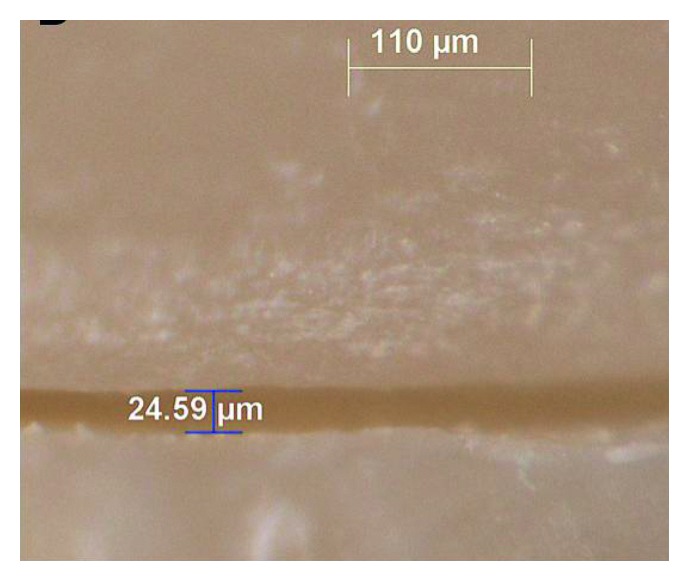
Measurement of marginal gap using a stereomicroscope (Leica MZ 16A, Leica Microsystems, Switzerland), Lava Ultimate inlay, distal.

For marginal gap measurements, inlays were placed, and to maintain the right position, a specially made clamp was applied. The marginal gaps were measured by one operator under a stereomicroscope (Leica MZ 16A, Leica Microsystems, Switzerland) using Leica Application Suite (Leica Microsystems, Switzerland) software, visually at 12 preselected locations, three on the mesial and 3 on the distal surfaces and 6 on the occlusal surface (3 occlusobuccal and 3 occlusolingual) of the MOD inlay (Figures 2 and 3).

### 2.4. Adhesive Placement of Restorations

All groups were cemented using Variolink N (Ivoclar Vivadent, Schaan, Liechtenstein). The inner surfaces of the restorations at EC, EL, and EP groups were etched with a 9.5% hydrofluoric acid gel (Bisco Porcelain Etchant, Bisco Inc., Illinois, USA) for 1 minute, rinsed with a water spray, and dried with oil-free air. According to the manufacturer's instructions, LU group was not etched. Then, a silane coupling agent (Monobond S, Ivoclar Vivadent, Schaan, Lichtenstein) followed by a light curing bonding agent (Heliobond, Ivoclar Vivadent, Schaan, Lichtenstein) was applied. Teeth were etched (30 s for enamel and 15 s for dentine) with 37% phosphoric acid. Tooth surfaces were conditioned with Syntac Primer, Adhesive, and Heliobond (Ivoclar Vivadent, Schaan, Lichtenstein). A dual-polymerising resin composite Variolink N (Ivoclar Vivadent, Schaan Lichtenstein) was placed on the inner surfaces of all restorations and cavity walls. Restorations were seated with finger pressure. Any excess cement was removed, and all surfaces of the restorations (occlusal, mesial, and distal) were then light-cured with a LED curing light wavelength of 470 nm and a power of 1200 mW/cm^2^ (Bluephase®, Ivoclar Vivadent, Schaan, Liechtenstein, 1200 mW/cm^2^) for 30 s. Excess cement was removed using polishing discs (Kerr OptiDisc, Bioggio, Switzerland) (Figure 1(c)).

All specimens were stored in distilled water at 37°C for 24 hours and then subjected to thermocycling at 5000 cycles in water baths between 5°C and 55°C. The dwell time at each temperature was 20 seconds, and the transfer time from one bath to the other was 5 seconds.

### 2.5. Fracture Strength Measurement

The teeth were subjected to axial compressive loading using a metal sphere of 5 mm diameter applied vertically and centered on the occlusal surface of the restoration at a crosshead speed of 0.5 mm/min in a universal testing machine (Lloyd Instruments LR 50K, AMETEK GmbH, Meerbusch, Germany). A thin plastic tape was placed on the surface of the ball to ensure a stable contact between the steel ball and tooth structure. The force (N) required to fracture the restoration was recorded for each specimen. The mode of fracture for each specimen was classified according to Burke [17] (Table 1).

The data obtained for the marginal gap were analyzed statistically using Kruskal–Wallis test and Mann–Whitney *U* test. Fracture resistance results were analyzed statistically by one-way ANOVA and Tukey HSD tests. The selected level of statistical significance was *p* < 0.05.

## 3. Results

The overall mean marginal gaps (*μ*m) for the three groups were EC = 33.54 (±13.83); LU = 33.77 (±17.35); EL = 34.23 (±16.62), and EP = 85.34 (±38.19). EC and LU marginal gaps were similar, and both were significantly less than EP (Table 1).

Statistical differences of surfaces mean marginal gaps (*μ*m) are shown at Table 2. Different surfaces' mean marginal gap values were not significantly different at EC and EP groups; however, at LU occlusobuccal and occlusolingual mean marginal gap calculations were significantly higher than mesial and distal surfaces values (*p* < 0.05) (Table 2).

**Figure 4:**
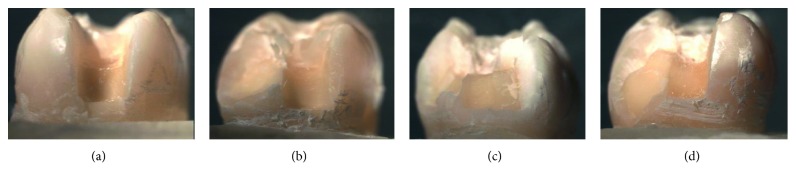
Fracture modes. (a) Type 1. (b) Type II. (c) Type III. (d) Type IV.

The means and standard deviations for the fracture strength of the test groups are shown in Table 3. One-way ANOVA analysis showed that there were statistically significant differences among the groups. The Tukey HSD test revealed that the control group (CO = 3959.00 N) showed significantly higher fracture strength values than the other groups (*p* < 0.05). No significant differences were observed between the fracture strength values of the groups restored with inlays (EC: 2408.00 N, LU: 2206.73, EL: 2573.27, and EP: 2879.53 N). The mode of fracture for each group is shown in Table 4. Fracture classifications of specimens were decided under a stereomicroscope (Leica MZ 16A, Leica) with inspection of all sides. Samples of each mode are given in Figure 4.

## 4. Discussion

Ceramic inlays has many advantages, which include high esthetic values and less tooth reduction compared to crowns or onlays; hence, more preservation of healthy tooth structures. Furthermore, with the development of CAD/CAM systems, the clinical process of placing inlays has become more efficient and convenient, which greatly improves the quality of the restoration and decreases the patient's visit time [18].

CAD-CAM technology has been introduced in the dental field to improve conventional impression techniques and manufacturing phases. Some studies show a higher marginal accuracy of restorations derived from an intraoral scanner in comparison to conventional impressions [19, 20]. Moreover, in this way, the dentist is able to check the preparation and can view the preparation simultaneously and discuss possible problems. On the other hand, scanning with a powder-free 3D measuring unit produces good values that are reliable, in particular, for single-tooth scans [21]. Conversely, an in vitro study showed that using powder before digital impression making with CEREC Omnicam resulted in significantly smaller marginal gap formations in crowns [22].

IPS Empress Esthetic restorations were fabricated using conventional two-step impression technique, whereas digital intraoral impressions were taken using CEREC Omnicam for IPS e.max CAD, Lava Ultimate, and IPS Empress CAD restorations.

Intact human teeth were used to evaluate and compare ceramic inlay restorations to obtain highly relevant clinical results. Also, adhesive cementation and bonding procedures were applied carefully to provide similar clinical outcomes [23]. The preparation was designed according to preparation guidelines for inlay restorations mentioned in the literature [19, 24]. Many variables in addition to ceramic type will influence the marginal gap value such as restoration fabrication method, location of the preparation, measuring techniques, number of measuring points, and tooth preparation design [25].

The direct measurement technique was used in the present study leaving the tooth intact and allowing for the reproducibility of measurements at different time intervals. This technique was used in several in vitro studies [26, 19, 27] and can therefore be considered as a well documented procedure. Direct measurement allows us to evaluate the marginal gaps from all angles and selected areas [26]. A large marginal discrepancy causes higher plaque index at restoration margins and periodontal problems. Thus, achieving a gap width below 100 *μ*m is desirable for clinically acceptable restorations [28]. The mean marginal discrepancies of inlay restorations of the present study were 85.34 *µ*m for IPS Empress Esthetic group, 33.54 *µ*m for IPS e.max CAD group, 34.23 *µ*m for IPS Empress CAD, and 33.77 *µ*m for Lava Ultimate group. These values were all within the clinically acceptable range. Additionally, conventional impression technique revealed wider marginal gap formation compared to digital impression technique CEREC Omnicam. The difference was attributed to thermal shrinkage of wax pattern and contraction, and also the expansion of ceramic material might have caused wider marginal gap formation. Hahn et al. [29] evaluated the marginal gap of IPS Empress inlays fabricated with conventional technique before cementation, and the results were lower (47 *µ*m) than the present study. However gap formations are both lower than clinically acceptable values. Guess et al. [30] reported 45 *µ*m marginal gap with conventional technique and similar to the present study CAD/CAM restorations showed lower marginal gap values. CAD/CAM-manufactured restorations produced with previous CEREC systems exhibited significantly larger marginal gap values than acceptable range [31, 32]. Nevertheless, the mean marginal gap values of CAD/CAM-fabricated restorations seem to decrease significantly by developments in systems [33, 34]. Reich et al. [35] reported that hot-pressing technique showed better marginal adaptation compared to CEREC 3D fabricated restorations. On the contrary, Guess et al. [30] found no significant difference between the marginal gap formation of CEREC 3D and hot-pressing technique fabricated restorations. However, similarly to the present study, some authors demonstrated marginal gap values between 36 and 43 *µ*m for CEREC 3 [19, 36, 37]. In the present study, it seems that the powder-free scans of teeth exhibited very low marginal gap values since the stereomicroscope measurements showed a high number of gap formations under 25 *µ*m.

Mean marginal gap values of surfaces showed differences only in Lava Ultimate group, but all values were under 38.48 *µ*m which is very lower than the clinically acceptable value.

In the literature, it is shown that the mean marginal gap values of heat-pressed and CAD/CAM-fabricated restorations increases significantly after cementation [30]. Beschnidt and Strub [38] reported that after cementation of ceramic restorations, marginal gap values increase 13-22 *µ*m whereas Wolfart et al. [39] suggested the change range is between 20 and 40 *µ*m. Therefore, the methodological differences for marginal adaptation measurements do not seem to affect clinically acceptable values in a drastic way.

In the present study, after adhesive cementation, thermal cycling was used to stimulate oral conditions. After simulated aging [30] of the inlay restorations, fracture strength test was applied using a metal sphere of 5 mm diameter to stimulate a molar cusp.

Teeth in posterior regions are subject to functional and parafunctional forces of varying magnitudes and directions [40]. Fractures in this region are a common problem which is affected by restoration type, fabrication methods, material structure, and finishing procedures [41–43].

The fracture strength was determined using a universal testing machine [24]. However, the universal testing machine did not reproduce oblique, torsional, and lateral shearing forces produced during chewing. During fracture strength tests, a single load increase was applied at a constant angle and the same area on the inlay received all forces. Nevertheless, masticatory forces are not constant but multidirectional and affect repeatedly larger surfaces [44]. The “vertical” or “compressive” nature of loading might be an oversimplification of the actual forces applied to the specimens.

The results of this study revealed that there were no significant differences between the fracture strength values of inlay groups; however, there were significant differences between inlay groups and control group. The unprepared molars achieved the highest mean fracture strength value of 3959.00 N. These results correlate with findings of other studies [24, 45]. Consistent with other studies, a large variability of fracture strength values was observed at fracture loading tests [46, 47]. Despite standard selection, storage, preparation of teeth, and milling the inlays in same conditions, it is impossible to control the distribution and length of internal cracks and flaws. IPS Empress glass ceramic has an increased dispersion of leucite caused by heat-pressing, and the high dispersion is expected to improve mechanical properties. Also, a more uniform dispersion of leucite may reduce the susceptibility of glass ceramics [48]. On the other hand, the milling process may cause a multitude of flaws which can act as potential cracking points. In contrast, a study reported a significantly higher fracture strength for CAD/CAM-produced leucite-reinforced glass ceramics than leucite-reinforced (IPS Empress) and lithium disilicate (IPS e.max Press) glass ceramics manufactured by the heat-pressed technique [49]. Similar to the present study, another investigation demonstrated no significant differences in fracture strength results of leucite-reinforced glass ceramics produced with CAD/CAM and heat-pressed technique [19]. Even though IPS e.max CAD has higher biaxial flexural strength values than Lava Ultimate [50], these results are not reflected in fracture strengths of restored teeth with MOD inlays.

Resin restorations are more fracture resistant than ceramics, especially in relatively thinner reconstruction [51]. Although the color stainability is a problem for resin nanoceramic restorations [52], the mechanical properties such as fracture strength, compressive strength, and enamel antagonist wear characteristics have some advantages over glass ceramics [9]. The resin nanoceramic Lava Ultimate elastic modulus [9] is very similar to natural human dentin's elastic modulus [53] values whereas IPS e.max CAD has higher structural behaviors [9]. In the present study, even though materials had different physical properties, inlay groups had similar fracture strength values. Distinctively, Bakeman et al. [54] reported that using a lithium disilicate glass ceramic for restorations significantly improved fracture strength compared to using a leucite-reinforced glass ceramic and ceramic thickness or ceramic materials that had no influence on fracture strength of posterior ceramic restorations. In contrast to our study, Yildiz et al. [55] reported that the fracture strength of heat-pressed ceramics was significantly higher than that of CAD/CAM onlays. The voids and cracks in the restorations produced by heat-pressed technique might have acted as cushioning effect and resulted as higher fracture strength values. In the present study, all restored groups exhibited similar fracture strength, attributed to the dimensions of the inlays produced. Since the occlusal thickness of MOD inlays produced were not larger than 2.8 mm, materials with different physical properties and fabrication methods may have demonstrated similar results.

Harada et al. [56] demonstrated that computer-milled composite and resin nanoceramic restorations showed adequate function for lost tooth structure. Besides, resin nanoceramic blocs showed superior fracture strength compared to composite blocs. They were able to be easily fabricated at a reduced cost and perhaps applied without the need for additional tooth reduction.

The fracture patterns of restored teeth were very consistent in this study. EC, EL, and EP groups showed mostly isolated fractures of the restorations; however, the LU group revealed restoration fractures involving a small tooth portion. All groups demonstrated similar percentages of unrepairable fractures with periodontal involvement.

Recently, CAD/CAM restorations' high success rate, color stability, minimal wear values, and acceptable marginal adaptation [9, 30, 52] allowed them to become a better alternative for direct restorations. Additionally, compared to indirect restorations, they avoid the costs of dental technicians and impression materials [57] and give the opportunity to finish in single appointment. CEREC Omnicam system with a practical use without powder application allows the dentists to apply chair-side ceramic restorations using optical impression of the tooth preparation. Furthermore, digital imaging provides archives for dentists while treating with highly sophisticated dental equipments.

## 5. Conclusions

Within the limitations of this study, CEREC Omnicam-fabricated inlays showed better marginal adaptation than inlays produced with heat-pressed technique. There was no statistical difference between fracture strength results of inlays fabricated with different materials and methods. Fracture patterns of materials used in this study mostly did not show severe fractures.

## Figures and Tables

**Table 1:** Results of the mean marginal gap. Standard deviation (SD) of the impression methods evaluated at each surface and overall mean marginal gap and SD of each impression method

Groups (*n*=15)	Inlay surface	Mean marginal gap (*µ*m)	Standard deviation (SD)	Overall mean marginal gap	Standard deviation (SD)
EC	Mesial	32.81	16.74	33.54^a^	15.83
IPS e.max CAD	Distal	36.05	20.54		
CEREC Omnicam	Occlusobuccal	32.69	12.69		
	Occlusolingual	32.60	12.15		

LU	Mesial	30.55	19.80	33.77^a^	17.35
Lava ultimate	Distal	29.11	18.92		
CEREC Omnicam	Occlusobuccal	38.48	15.97		
	Occlusolingual	36.93	12.44		

EL	Mesial	32.71	18.94	34.23^a^	17.67
IPS empress CAD	Distal	31.94	18.51		
CEREC Omnicam	Occlusobuccal	36.82	17.22		
	Occlusolingual	35.45	16.03		

EP	Mesial	88.64	37.51	85.34^b^	38.19
IPS empress esthetic	Distal	86.80	44.29		
CEREC Omnicam	Occlusobuccal	84.16	32.67		
	Occlusolingual	81.78	38.35		

Values with the same superscript letter are not significantly different (*p* < 0.001). ^a^Same superscript letters in same column indicates no significant difference (*p* > 0.05). ^b^Different superscript letters in same column indicates significant difference (*p* < 0.001).

**Table 2:** Cross table and comparison of different surfaces marginal gap results.

	EC				LU				EL				EP			
	IPS e.max CAD				Lava Ultimate				IPS Empress CAD				IPS Empress Esthetic			
	CEREC Omnicam				CEREC Omnicam				CEREC Omnicam				CEREC Omnicam			
	Mesial	Distal	Occlusobuccal	Occlusolingual	Mesial	Distal	Occlusobuccal	Occluso lingual	Mesial	Distal	Occlusobuccal	Occlusolingual	Mesial	Distal	Occlusobuccal	Occlusolingual
Mesial	—	0.696	0.608	0.589	—	0.439	0.008^s^	0.001^s^	—	0.537	0.438	0.379	—	0.526	0.564	0.260
Distal	0.696	—	0.907	0.910	0.439	—	0.004^s^	0.001^s^	0.669	—	0.715	0.744	0.526	—	0.875	0.597
Occlusobuccal	0.608	0.907	—	0.990	0.008^s^	0.004^s^	—	0.713	0.622	0.652	—	0.538	0.564	0.875	—	0.300
Occlusolingual	0.589	0.910	0.990	—	0.001^s^	0.001^s^	0.713	—	0.511	0.685	0.687	—	0.260	0.597	0.300	—

Values with the “s” letter are significantly different according to the Mann–Whitney U analysis. (*p* < 0.05).

**Table 3:** Mean fracture resistance values, standard deviations, and statistical categories of all experimental groups (*n*=15).

Groups	Material	Fracture strength mean values (N)	Standard deviation (SD)
CO	Control	3959.00^a^	1279.79
EC	IPS e.max CAD	2408.00^b^	607.97
LU	Lava Ultimate	2206.73^b^	675.16
EL	IPS Empress CAD	2573.27^b^	644.73
EP	IPS Empress Esthetic	2879.53^b^	897.30

Groups with different superscript letters are statistically significantly different according to the Tukey HSD test (*p* < 0.05). ^a^Different superscript letters in same column indicates significant difference (*p* < 0.001). ^b^Same superscript letters in same column indicates no significant difference (*p* > 0.05).

**Table 4:** Fracture modes of restored specimens according to Burke [17].

Mode of failure	EC	LU	EL	EP
	IPS e.max CAD	Lava Ultimate	IPS Empress CAD	IPS Empress Esthetic
	CEREC Omnicam	CEREC Omnicam	CEREC Omnicam	CEREC Omnicam
I	6 (40.0%)	2 (13.3%)	5 (33.3%)	5 (33.3%)
II	4 (26.7%)	6 (40.0%)	5 (33.3%)	5 (33.3%)
III	- (0%)	3 (20%)	2 (13.3%)	1 (6.7%)
IV	5 (33.3%)	4 (26.7%)	3 (20%)	4 (26.7%)

Mode I: isolated fracture of the restoration; mode II: restoration fracture involving a small tooth portion; mode III: fracture involving more than half of the tooth, without periodontal involvement; mode IV: fracture with periodontal involvement.
